# Identification of key anti-glycation polyphenols in Sakura through metabolic profiling and *in vitro* assessments

**DOI:** 10.1016/j.fochx.2025.102416

**Published:** 2025-03-25

**Authors:** Yuhang Zhu, Zhangtie Wang, Xiang Li, Siyu Chen, Daoxin Dai, Weihu Li, Binhai Shi, Baolong Wang, Guoliang Jie, Baiyi Lu

**Affiliations:** a College of Biosystems Engineering and Food Science, Key laboratory for Quality Evaluation and Health Benefit of Agro-Products of Ministry of Agriculture and Rural Affairs, Key Laboratory for Quality and Safety Risk Assessment of Agro-Products Storage and Preservation of Ministry of Agriculture and Rural Affairs, Zhejiang University, Hangzhou, China; b Shiseido China Innovation Center, Shanghai, China; c Yangzhou China and Yeal Food Co., Ltd., Yangzhou, China; d Huangshan GreenXtract Co., Ltd., Huangshan, China

**Keywords:** Anti-glycation, Sakura, Polyphenols, Metabolomics, Simulated digestion

## Abstract

Sakura, a traditional edible flower, has been investigated for its anti-glycation potential; however, the bioactive components remain unclear. Polyphenols are recognized for their exceptional anti-glycation properties. In this study, main polyphenols were identified from seven varieties through metabolomics. 5-O-caffeoylquinic acid (5-OCA), 3-O-caffeoylquinic acid (3-OCA), and caffeic acid were confirmed as key agents through multivariate analysis, which was further validated by anti-glycation assessments. The concentrations of these polyphenols in *Cerasus serrulata ‘Kanzan’* (CK) were 2.52 ± 0.08, 3.31 ± 0.18 and 2.38 ± 0.15 mg/g, respectively, contributing to its superior AGEs inhibition ratio (100 μg/mL) of 76.11 ± 0.40 %. Simulated digestion revealed that isomerization between 5-OCA and 3-OCA occurred during the gastric phase. Both compounds were metabolized to caffeic acid in the intestinal phase, contributing to a sustained anti-glycation activity of 64.74 ± 1.11 %. Overall, our study provides a theoretical basis for the development of Sakura-based functional foods targeting anti-glycation.

## Highlights

• Anti-glycation properties of seven Sakura varieties were assessed
• Most abundant polyphenols in seven Sakura varieties were identified *via* metabolomics
• Multivariate analysis revealed 5−/3-OCA and caffeic acid as key anti-glycation agents
• Isomerization between 5-OCA and 3-OCA occurred in gastric phase
• 5-OCA and 3-OCA were eventually metabolized to caffeic acid during digestion

## 1 Introduction

Protein glycation, commonly referred to as the Maillard reaction, is a non-enzymatic process characterized by the condensation of amino groups in protein side chains with carbonyl groups of reducing sugars (Sun et al., 2024). This reaction plays a crucial role in the formation of stable covalent adducts, recognized as advanced glycation end products (AGEs) (Gu et al., 2024). During the glycation process, a reversible Schiff base is initially formed through nucleophilic addition, which subsequently rearranges to yield Amadori products. These Amadori products are then converted into reactive α-dicarbonyls, such as methylglyoxal and glyoxal, ultimately leading to the formation of AGEs (Li et al., 2014; Sun et al., 2018). AGEs can originate exogenously from dietary sources or be synthesized endogenously in various organs, tissues, and body fluids (Zhang et al., 2020). The ongoing formation and accumulation of AGEs *in vivo* are closely linked to the pathogenesis of chronic and age-related diseases, such as diabetic complications, cardiovascular diseases and Alzheimer's disease, through structural modification of functional proteins in the brain, skin and blood vessels (Rojas et al., 2024; Wang, Jiang, & Zhao, 2024; Zgutka et al., 2023). Although several synthetic compounds, such as aminoguanidine (AG), have been developed and shown to possess promising anti-glycation effects, their potential side effects present unavoidable health risks, thereby limiting their application as anti-glycation therapeutics (Borg & Forbes, 2016; Brownlee, 1992). The urgent need for effective and safe anti-glycation agents has promoted research into the potential of various phytochemicals derived from natural plants as inhibitors of AGEs formation (Chinchansure et al., 2015; Q. Song et al., 2021). Notably, dietary polyphenols, a widespread class of phytochemicals, such as resveratrol, ellagic acid, and procyanidins, have been extensively investigated and shown to possess significant anti-glycation activities by inhibiting reactive oxygen species, trapping dicarbonyl compounds, and disrupting AGE-induced crosslinking (Chang et al., 2022; Yue et al., 2023; Zheng et al., 2022).

With the increasing consumption of vegetable leaves, roots, fruits, and seeds, there is a growing interest in edible flowers (Chen et al., 2020). Edible flowers have a long history of use as traditional food additives and medicinal agents in both Asian and European cultures (Loizzo et al., 2016). An increasing number of studies indicate that edible flowers are abundant in phytochemicals, including phenolic acids and flavonoids, which contribute to their potential health benefits (Amrouche et al., 2022; Wang, Zhu, et al., 2024). *Cerasus* flowers, commonly known as Sakura, are traditional edible flowers widely distributed across East Asian countries. In Japan, Sakura not only symbolize the arrival of spring but are also extensively used in cuisine, including salted Sakura, Sakura tea, and Sakura sake (Moriuchi & Basil, 2019). China is one of the original regions for Sakura cultivation. Although the history of consuming Sakura in China is relatively short, the popularization of Sakura culture has led to an increase in traditional pastries such as Sakura rice cakes and Sakura jam in recent years. It is noteworthy that CK was officially recognized as a food raw material in China in 2022, which has opened up substantial economic opportunities within the food industry (Suo et al., 2023). Recently, researchers have investigated the health benefits and applications of Sakura, revealing that functional components such as flavonoids and polysaccharides play a crucial role in anti-oxidation, anti-inflammation, anti-tumor, and anti-aging processes (Xiang et al., 2023). Furthermore, cinnamoyl and flavonol glucosides extracted from Sakura have been demonstrated to inhibit the formation of AGEs and prevent AGE-induced fibroblast apoptosis (Shimoda et al., 2011). However, the phytochemical profiles of different Sakura varieties exhibit considerable variations, and few studies have examined the correlations between their composition and bioactivity. Polyphenols are recognized as a significant class of bioactive compounds in edible flowers, noted for their capability to inhibit the formation of AGEs and mitigate the detrimental effects associated with AGE accumulation. Nonetheless, the analysis and identification of key polyphenols in Sakura, as well as the variations in their content among major varieties, remain insufficiently understood. Furthermore, the metabolic fate of polyphenols derived from Sakura during digestion, which is critical for evaluating their bio-accessibility and sustained anti-glycation efficacy, remains unexplored.

In this study, the anti-glycation activities of seven Sakura varieties were evaluated. Among these, the CK variety, which exhibited the highest total phenolic content (TPC), demonstrated the strongest anti-glycation activity. Based on existing literature, it can be hypothesized that polyphenols played a crucial role in the anti-glycation activity of Sakura (Yeh et al., 2017). Metabolomic analysis identified the polyphenols with the highest relative content across the seven varieties. Multivariate data analysis revealed a strong positive correlation between the contents of 5-OCA, 3-OCA, and caffeic acid and their anti-glycation activity. Furthermore, HPLC quantitative analysis confirmed that the CK variety, which exhibited the superior anti-glycation activity, also contained the highest levels of these three compounds. *In vitro* assessments further validated that the AGEs inhibition ratios of 5-OCA, 3-OCA, and caffeic acid were significantly higher than those of other polyphenols with relatively high contents. Therefore, 5-OCA, 3-OCA, and caffeic acid were identified as the key anti-glycation polyphenols in Sakura. The transformation and metabolism processes of these compounds were further analyzed through simulated *in vitro* digestion. This research lays a crucial foundation for identifying the most promising Sakura variety for inhibiting the formation of AGEs and elucidating the key polyphenols and their metabolic pathways during digestion. These findings aim to establish a theoretical basis for the development of Sakura-based functional foods or pharmaceuticals targeted on anti-glycation.

## 2 Materials and methods

### 2.1 Chemicals and reagents

*Cerasus serrulata ‘Kanzan’* (CK), *Cerasus yedoensis (Matsum.) T.T. Yu et Li* (CT), *Cerasus sargentii (Rehder) Eremin, Yushev & L. N. Novikova* (CE), *Cerasus speciosa (Koidz.) H. Ohba* (CH), *Cerasus campanulata (Maxim.) Yü et Li* (CY), *Cerasus serrulata (Lindl.) G. Don* (CG) and *Cerasus serrula (Franch.) Yü et Li* (CL) were provided by Yangzhou China and Yeal Food Co., Ltd. (Yangzhou, China). The appearance and supplier information for seven Sakura varieties were listed in Table S1. All chemicals, solvents, and standards of analytical reagent grade were sourced from commercial suppliers, and were either used as received or dried according to standard procedures. The standards utilized for metabolomic and quantitative analysis included 5-OCA (CAS Registry Number 906–33-2), 3-OCA (CAS Registry Number 327–97-9), kaempferol-3-O-glucoside (CAS Registry Number 480–10-4), quercetin-3-O-glucoside (CAS Registry Number 21637–25-2), kampferol-3-O-rutinoside (CAS Registry Number 17650–84-9), protocatechuic acid (CAS Registry Number 99–50-3), succinic acid (CAS Registry Number 110–15-6) and caffeic acid (CAS Registry Number 331–39-5), which were purchased from Chengdu Alfa Biotechnology Co., Ltd. (Chengdu, China). Acetonitrile, methanol, formic acid and phosphoric acid (HPLC grade) were obtained from Sigma-Aldrich (Steinhemin, Germany). Folin-phenol reagent, BSA (BR, 98 %) and PC-300 were obtained from Solarbio Science & Technology Co., Ltd. (Beijing, China). α-amylase (from porcine pancreas, 1500 U/mL), pepsin (from porcine gastric mucosa, 20,000 U/mL), pancreatin (from porcine pancreas, 800 U/mL) and amyloglucosidase (from *Aspergillus niger*, 400 U/mL) were acquired from Aladdin Reagents Co., Ltd. (Shanghai, China). All other chemicals were provided by Sinopharm Chemical Reagent Co. Ltd. (Shanghai, China).

### 2.2 Extract preparation

The extraction procedure was conducted following a previous method (Chen et al., 2023). Sakura samples were extracted using pure water (1:40, *w*/*v*) through an ultrasonic-assisted process (40 °C, 500 W) for 1 h. After filtration, the solvent was removed using rotary evaporation (RE-2000 A rotary evaporator, Shanghai Yarong Biochemistry Instrument Factory). The concentrated extracts were then lyophilized (HXLG-12-50D vacuum freeze drier, ShanghaiHuxishiye Co., Ltd.) and subsequently stored at −80 °C for further analysis.

### 2.3 Determination of total phenolic contents

The content of total phenolic content was quantified by the previous Folin-Ciocalteu method with some modifications (Xiong et al., 2014). Briefly, the diluted sample solution (0.5 mL) was mixed with Folin- Ciocalteu's reagent (0.1 mL) before incubating for 6 min. 1 mL of Na_2_CO_3_ (7 %, w/v) was then added, and final volume of the mixture was adjusted to 6 mL with deionized water. The thermostatic reaction was processed at 37 °C for 90 min before measuring the absorbance at 760 nm. The TPC was calculated as milligram of 3-OCA equivalents per gram dry weight (mg 3-OCAE/g DW) using a 3-OCA standard curve (0–100 μg/mL).

### 2.4 Anti-glycation assessment in BSA-Glu/Fru model

The inhibition of AGEs formation was evaluated using a BSA-Glu/Fru model, which was adapted from our previous work with slight modifications (Wang, Zhu, et al., 2024). The BSA-Glu/Fru solution was prepared in phosphate buffer (50 mM, pH 7.4) containing BSA (20 mg/mL), glucose/fructose (90 mg/mL or 0.5 mM) and 0.1 % (*v*/v) PC-300. For the assessment of Sakura extracts, the resulting protein solutions were incubated at 37 °C in the dark, with or without floral extracts prepared as previously described, at concentrations of 100, 200 and 300 μg/mL. The assessment of polyphenol standards (10–800 μM) was conducted using the same procedure. AG (1–10 mM) was used as positive control and the model without sugars served as a blank control. After 7-day incubation, the formation of total fluorescent AGEs and pentosidine were analyzed by measuring the fluorescence intensity at a wavelength of λ_ex_/λ_em_ = 340/420 nm and 335/385 nm, respectively.

Inhibition of the formation of fluorescent AGEs/pentosidine (%) was calculated by the following equation:

$$\text{Inhibition}\;\left(\%\right)=\left(1-\frac{F_{\text{sample}}-F_{\text{background}}}{F_{\text{control}}-F_{\text{background}}}\right)\times 100$$

### 2.5 Metabolic profiling by UPLC-MS/MS

The metabolic profile of seven Sakura varieties was analyzed using UPLC-MS/MS. Sample solution was prepared by dissolving extract in 20 % dimethyl sulfoxide (2 mg/mL). After centrifuging at 10,000 rpm for 2 min, the supernatant was collected for further analysis. The UPLC system (Thermo Fisher Scientific, Hemel Hempstead, UK) was equipped with a UHPLC BEH C_18_ column (2.1 mm × 100 mm, 1.7 μm). Mobile phase A consisted of 0.1 % formic acid, while mobile phase B contained 0.1 % formic acid in acetonitrile. The column temperature, flow rate, and injection volume were maintained at 35 °C, 0.4 mL/min, and 10 μL, respectively. The elution program was as follows: 0–3 min, 95 %–75 % A; 3–4 min, 75 %–35 % A; 4–10 min, 35 % A; 10–10.1 min, 35 %–95 % A; 10.1–13 min, 95 % A. Both positive and negative ion detection modes were employed and data were recorded over a mass-to-charge ratio (*m*/*z*) range of 100–1000. The instrumental parameters were set as described in our previous study (Chen et al., 2023). Polyphenols were identified based on molecular mass, retention times, MS fragments with *mzCloud* database *via* Compound Discoverer software (Thermo Fisher Scientific, Hemel Hempstead, UK) and further confirmed with commercially available standards. Semi-quantification was performed for the remaining polyphenols and metabolites. XCalibur 3.0 software (Thermo Fisher Scientific, Hemel Hempstead, UK) was used for system control and data processing.

### 2.6 *In vitro* digestion

*In vitro* simulated digestion of Sakura extracts was conducted according to previously established method with slight modifications (Minekus et al., 2014). Initially, simulated salivary fluid (SSF), gastric fluid (SGF) and intestinal fluid (SIF) were prepared in accordance with the INFOGEST *in vitro* gastrointestinal digestion protocol (Brodkorb et al., 2019). For the oral digestion phase, 30 mg of the extract was dissolved in 5 mL of distilled water. The solution was mixed with 3.5 mL of SSF, 0.5 mL of α-amylase (1500 U/mL), 25 μL of CaCl_2_ solution (0.3 M) and 975 μL of distilled water. The resultant mixture was incubated in a thermostatic water bath at 37 °C with shaking at 120 rpm for 2 min. Subsequently, 7 mL of salivary solution, 4.2 of mL SGF, 1.4 mL of pepsin (20,000 U/mL), 3.5 μL of CaCl_2_ (0.3 M) and 1.2565 mL of distilled water were combined and the pH was adjusted to 3.0 with 0.14 mL of HCl (1 M). The resultant mixture was then incubated in a thermostatic water bath at 37 °C with shaking at 120 rpm for 2 h for gastric digestion. Intestinal digestion proceeded with a combination of 9 mL of gastric solution, 2.7 mL of SIF, 2.25 mL of pancreatin (1600 U/mL), 2.25 mL of amyloglucosidase (800 U/mL), 2.25 mL of 1.125 mL bile salt (0.16 M), 18 μL of CaCl_2_ (0.3 M) and 589.5 μL of distilled water. After adjusting the pH to 7.0 with 67.5 mL NaOH (1 M), the mixture was incubated in a thermostat water bath at 37 °C with shaking at 120 rpm for an additional 2 h. The headspace of the bottle was flushed with N_2_ in each stage of the reaction to remove air. At the end of each phase, aliquots were collected and centrifuged at 4 °C (12,000 g, 5 min), the supernatant was then lyophilized and stored at −20 °C until further analysis.

### 2.7 Quantitative analysis of the primary polyphenols *via* HPLC-PDA

Before analysis, floral extracts or digested mixtures were diluted with 50 % methanol and filtered using a 0.22 μm Millipore filter membrane to remove impurities. Quantitative analyses of 5-OCA, 3-OCA and caffeic acid in different samples before, during, and after digestion were conducted using an Agilent 1200 series liquid chromatograph equipped with a quaternary gradient pump, an autosampler and a PDA detector. The system utilized an Agilent C_18_ column (5 μm, 4.6 × 250 mm; Agilent Technologies, Santa Clara, CA, USA) maintained at 30 °C. Aqueous 0.1 % phosphoric acid (solvent A) and acetonitrile (solvent B) were employed at a flow rate of 1.0 mL/min. The gradient was established as follows: 0–9 min, 91 %–89 % A; 9–14 min, 89 %–85 % A; 14–16 min, 85 %–83 % A; 16–17 min, 83 %–10 % A; 17–20 min, 10 % A; 20–21 min, 10 % -91 % A; 21–23 min, 91 % A. Peaks were identified by comparing their retention times and quantified through external standardization with calibration curves using commercially available standards, with their peak areas referenced to a calibration curve obtained under 330 nm.

### 2.8 Statistical analysis

All experiment were independently repeated in triplicate, with results expressed as the mean ± standard deviation. The *p* values at<0.05 were considered significant, as assessed *via* one-way analysis of variance (ANOVA) using SPSS Statistics 24.0 (IBM Corp., Chicago, IL, USA). Multivariate analyses, including principal component analysis (PCA) and hierarchical clustering for heatmap generation, were conducted using OriginPro 2024 (OriginLab Corp., Northampton, MA, USA). Data visualization was performed with GraphPad Prism v9.0 (GraphPad Software, La Jolla, CA, USA), while chemical structures were illustrated using ChemDraw Professional 22.2 (PerkinElmer Inc., Waltham, MA, USA).

## 3 Results and discussion

### 3.1 Measurement of TPC in seven Sakura varieties

**Fig. 1:**
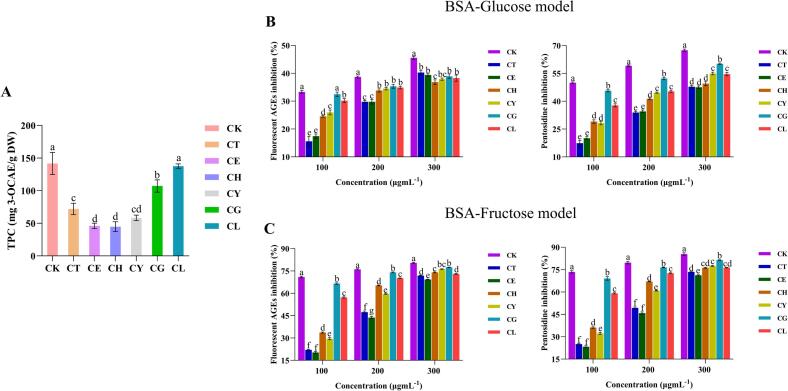
**(A)** The TPC results of seven Sakura extracts. **(B)** The inhibitory effects of seven Sakura extracts on the total fluorescent AGEs and pentosidine in the BSA-Glu model. **(C)** The inhibitory effects of seven Sakura extracts on the total fluorescent AGEs and pentosidine in the BSA-Fru model.

The accumulation of AGEs *in vivo* is closely associated with the development of oxidative stress, low-grade chronic inflammation, which contribute to the progression of various diseases, including diabetes, cardiovascular diseases, and aging-related disorders (Khan et al., 2020). In recent years, polyphenols have been extensively studied for their remarkable inhibitory effects on the formation of AGEs, owning to their antioxidant, anti-inflammatory, and anti-aging activities, (Song et al., 2021; Yeh et al., 2017). Notably, polyphenols have also been confirmed as the primary anti-glycation components found in natural resources such as medicinal plants and edible flowers (Yeh et al., 2017). Based on previous studies, polyphenolic components were considered as the key targets in our research. Accordingly, the TPC of seven Sakura varieties was initially assessed. As shown in Fig. 1A, the TPC of CK, CT, CE, CH, CY, CG and CL were determined as 141.64 ± 16.97, 71.95 ± 8.75, 46.04 ± 4.20, 58.37 ± 4.16, 107.17 ± 9.43 and 137.61 ± 3.40 3-OCAE/g DW, respectively. The TPC of CK, CG and CL was significantly higher than that of the other varieties (*p* < 0.05).

### 3.2 Anti-glycation assessment of seven Sakura varieties

The anti-glycation effects of seven Sakura extracts were evaluated using BSA-Glu and BSA-Fru models, both of which are widely applied to simulate all stages of protein glycation (Park & Lee, 2021). Pentosidine, one of the most common fluorescent AGEs in various tissues, is extensively studied as a biomarker for monitoring the process of the formation of AGEs (André et al., 2018; Sun et al., 2018). Consequently, the anti-glycation effects were assessed by evaluating the inhibition ratios of both AGEs and characteristic pentosidine formation. Fig. 1B and C illustrated the inhibition ratios of the seven Sakura extracts on fluorescent AGEs and pentosidine at three different concentrations. In the BSA-Glu model, CK and CG exhibited the highest inhibition ratio of 32.69 ± 0.54 % and 31.62 ± 0.79 %, respectively, with no significant difference in total fluorescent AGEs at a concentration of 100 μgmL^−1^ (*p* > 0.05). Notably, CK demonstrated the highest inhibition ratio of 38.68 ± 0.35 % and 45.54 ± 0.49 % at concentrations of 200 and 300 μgmL^−1^, respectively. The inhibition ratios of the seven Sakura extracts on pentosidine mirrored the trends observed for AGEs, with CK presenting the highest inhibition ratios of 49.99 ± 0.45 %, 59.12 ± 0.39 % and 67.49 ± 0.63 % at concentrations of 100, 200 and 300 μgmL^−1^, respectively. As depicted in Fig. 1C, CK also exhibited the highest inhibition ratios of 70.89 ± 0.52 %, 76.11 ± 0.40 % and 80.40 ± 0.37 % on AGEs at three different concentrations in the BSA-Fru model, which were significantly higher than those observed in the in BSA-Glu model (*p* < 0.05). This difference may be attributed to the greater reactivity of fructose towards BSA in forming AGEs through non-enzymatic glycation reactions compared to glucose, resulting in the synthesis of more AGEs in the control group. Furthermore, CK displayed the highest inhibition ratios of 73.48 ± 0.80 %, 79.69 ± 0.82 % and 85.44 ± 0.84 % on pentosidine at three concentrations in the BSA-Fru model (*p* < 0.05). Consequently, CK exhibited the most remarkable anti-glycation efficacy when compared to the other six common varieties, based on the combined experimental results from both BSA-Glu and BSA-Fru models. It can be hypothesized that the superior anti-glycation efficacy of CK is related to its high phenolic content, which has already been assessed.

### 3.3 Metabolic profiling of seven Sakura varieties

**Fig. 2:**
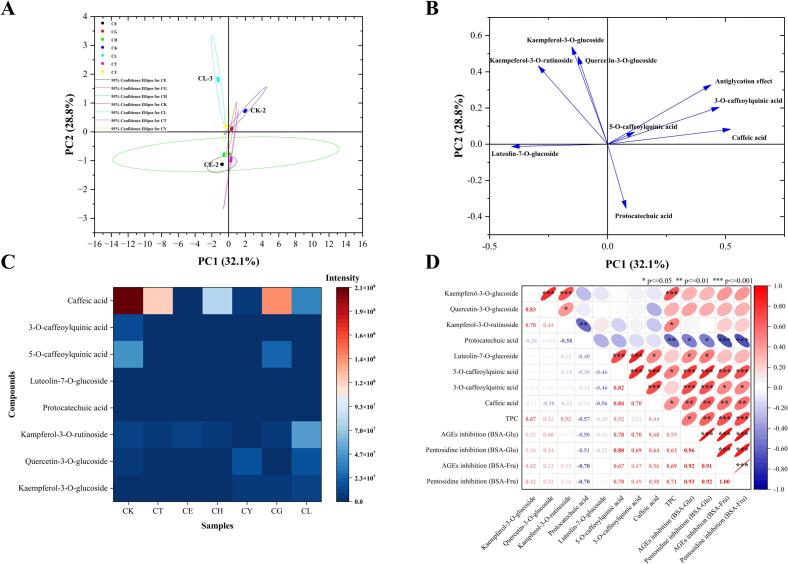
Multivariate data analysis on metabolic profiling targeted on polyphenols and anti-glycation activities of seven Sakura varieties. **(A)** Scores plot. **(B)** Loading plot. **(C)** Heat map of relative content of polyphenols in seven varieties. **(D)** Heatmap correlation diagram of polyphenols, TPC and anti-glycation effects.

Next, our study aimed to identify the main polyphenols through metabolomics using UPLC-MS/MS, and to analyze the differences in polyphenolic compositions across seven Sakura varieties. As shown in Table 1, after matching with *mzCloud* database and MS data of the available standards, the most abundant polyphenols were identified from seven varieties. These polyphenols exhibited relative peak intensities ≥1 × 10^5^ as analyzed by metabolomic profiling. The discrepancies between the theoretical and experimental mass of the parent ion were less than 10 ppm, indicating a high level of accuracy in the metabolomic analysis. Heatmap analysis was conducted to visualize the content differences of eight identified polyphenols with high relative content in seven Sakura varieties using semi-quantitative methods based on their peak intensities **(**Fig. 2C). Although this method did not yield accurate quantitative result for each polyphenol, it clarified the relative amounts and variation trends of each polyphenol across different Sakura extracts. As anticipated, the results aligned with those of the TPC analysis. The relative amounts of 3-OCA, 5-OCA and caffeic acid in CK were the highest among seven varieties. Notably, the relative amounts of caffeic acid in CK, CT and CG surpassed those in the other varieties. Kaempferol-3-O-glucoside, caffeic acid and 5-OCA were undetected in CE, where the levels of other polyphenols were also relatively low except for succinic acid, which accounted for the comparatively poor anti-glycation activity. Quercetin-3-O-glucoside was not detected in CH. It was noteworthy that the peak intensity of caffeic acid was significantly higher than that of any other compound across seven varieties. However, subsequent quantitative analysis *via* HPLC revealed that the content of 3-OCA was even higher than that of caffeic acid in CK, CT, CE and CL. This discrepancy may be attributed to the more efficient detection of caffeic acid in positive ion mode compared to the negative ion mode used for the detection of the other seven polyphenols, as indicated by the metabolomics analysis (Lucci et al., 2017).

**Table 1:** Identification of polyphenols with high relative abundances in seven Sakura varieties by UPLC-MS/MS.

Compound name	Molecular formula	Ion mode	Retention time (min)	Ion mass			MS^2^ fragment ions
				**Theoretical**	**Experimental**	**Error (ppm)**	
protocatechuic acid	C_7_H_6_O_4_	−	2.16	153.0193	153.0192	0.7	153.0067, 109.0196
5-O-caffeoylquinic acid	C_16_H_18_O_9_	−	2.21	353.0878	353.0848	8.5	353.0625, 191.0408, 179.0381, 135.0451
caffeic acid	C_9_H_8_O_4_	+	2.34	181.0495	181.0509	7.7	163.0400, 145.0294, 135.0450, 117.0346
luteolin-7-O-glucoside	C_21_H_20_O_11_	−	2.55	447.0933	447.0976	9.6	447.0625, 284.0120,255.0114
3-O-caffeoylquinic acid	C_16_H_18_O_9_	−	2.62	353.0878	353.0849	8.2	353.0632, 191.0409, 85.0271
quercetin-3-O-glucoside	C_21_H_20_O_12_	−	3.88	463.0882	463.0845	8.0	463.0709, 300.0157, 271.0144, 255.0199
kampferol-3-O-rutinoside	C_27_H_30_O_15_	−	4.06	593.1512	593.1559	7.9	593.1281, 285.0289, 255.0198, 227.0257
kaempferol-3-O-glucoside	C_21_H_20_O_11_	−	4.24	447.0933	447.0960	6.0	447.0765, 284.0216, 255.0199, 227.0257

### 3.4 Identification of key polyphenols contributing to anti-glycation activity

The anti-glycation activities of seven Sakura samples have been confirmed, and eight of the most abundant polyphenols among them have been identified. However, the key compounds that contributed most significantly to the anti-glycation effect remain to be elucidated. To address this, multivariate data analysis was performed by PCA and correlation analysis to clarify the differences among Sakura varieties and to identify the key bioactive polyphenols for anti-glycation. PCA is a widely utilized unsupervised technique that transforms correlated variables into a set of uncorrelated indicators, thereby reducing data dimensionality and extracting principal components that account for the majority of the variance in the dataset (Wei et al., 2024). Typically, the outcomes of PCA consist of two components: the score plot and the loading plot. The score plot visually illustrates the similarities or differences among samples, while the loading plot reveals the contribution of individual variables to the discrimination between categories (Pacholczyk-Sienicka et al., 2024). Fig. 2A and B illustrated the score and loading plots of PCA derived the data acquired from metabolic profiling, TPC and anti-glycation assessments. In Fig. 2A, the score plot revealed seven distinct clusters, accounting for 60.9 % of the cumulative variance. PC1 accounted for the largest portion of the variance, explaining 32.1 %, while PC2 accounted for an additional 28.8 %. The sample CK and CG were positioned in the first quadrant with positive scores for both PC1 and PC2, which corresponded with relatively high anti-glycation activities among the seven different Sakura varieties. A clear separation was noted for CH and CT, which were found in the second quadrant with positive scores for PC1 and negative scores for PC2. In the third quadrant, CE and part of CH were distinctly separated, displaying negative scores for both PC1 and PC2. Conversely, CY and CL were positioned in the fourth quadrant, displaying negative scores for PC1 and positive scores for PC2, demonstrating good differentiation. The segregation observed among these seven Sakura varieties is primarily attributed to the spectral differences in polyphenolic acids and their varying inhibitory effects on fluorescent AGEs, as indicated by the loading variables in the PCA analysis. As depicted in the loading plot (Fig. 2B), PC1 was positively correlated with 3-OCA, 5-OCA, caffeic acid, protocatechuic acid and anti-glycation effects, while it was negatively correlated with kaempferol-3-O-glucoside, quercetin-3-O-glucoside, kaempferol-3-O-rutinoside and luteolin-7-O-glucoside. PC2 showed a positive correlation with all polyphenolic components except protocatechuic acid and luteolin-7-O-glucoside. It was evident that 3-OCA, 5-OCA, caffeic acid, protocatechuic acid and anti-glycation effects were situated in the first quadrant, indicating that these three polyphenols exhibited the closest positive correlations with anti-glycation effects. Consequently, the PCA analysis not only facilitated the differentiation of various Sakura varieties to a certain extent, but also established the relevant classification criteria based on the distribution of loading variables. Additionally, it provided a preliminary identification of the most critical polyphenols responsible for anti-glycation activities.

To further corroborate that 5-OCA, 3-OCA and caffeic acid were the dominant anti-glycation polyphenolic components in Sakura extracts, the heatmap correlations illustrated in Fig. 2D emphasized the relationships among eight identified polyphenols, TPC and anti-glycation effects in BSA-Glu/Fru models. As anticipated, TPC exhibited a significant positive correlation with the inhibition of AGEs (*p* < 0.05) and pentosidine (*p* < 0.01) in the BSA-Glu model, as well as with the inhibition of AGEs and pentosidine (*p* < 0.001) in the BSA-Fru model. This finding was consistent with previous studies indicating that polyphenolic components played a dominant role in anti-glycation activities of edible flowers. Kaempferol-3-O-glucoside demonstrated a strong correlation with TPC (*p* < 0.001), while kaempferol-3-O-rutinoside, 5-OCA and caffeic acid also exhibited positive correlations with TPC (*p* < 0.05). However, no significant correlation was observed between kaempferol-3-O-glucoside /kaempferol-3-O-rutinoside and anti-glycation effects. Based on these results, it was reasonable to conclude that CK exhibited the strongest inhibitory effects on AGEs and pentosidine among the seven varieties studied. Among the eight polyphenols, only 5-OCA, 3-OCA and caffeic acid were significantly correlated with the inhibitory effects of AGEs and pentosidine in both BSA-Glu and BSA-Fru models. Notably, 5-OCA showed the strongest positive correlation (*p* < 0.001) with anti-glycation effect in both models, further validating the preliminary hypothesis derived from PCA analysis. Additionally, it was interesting to note that 5-OCA, 3-OCA, and caffeic acid were significantly correlated with one another (*p* < 0.001). This correlation was particularly noteworthy given that 5-OCA and 3-OCA are isomers with similar structures but differing positions of the ester groups, while caffeic acid is the metabolite resulting from the hydrolysis of 5-OCA and 3-OCA (Cortez et al., 2024).

**Fig. 3:**
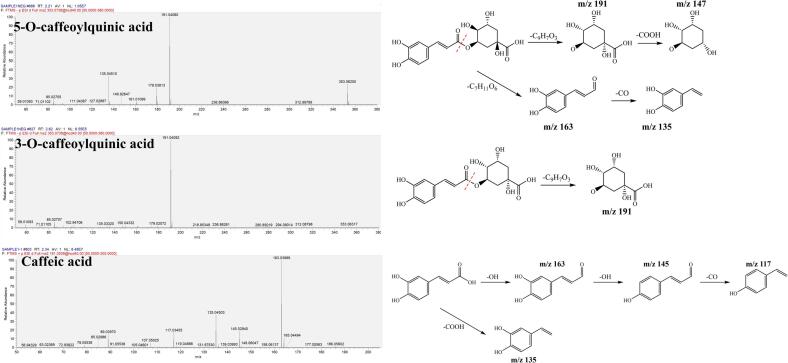
MS/MS spectra and fragment cleavage patterns of 3-OCA, 5-OCA and caffeic acid.

Based on the MS/MS spectra presented in Fig. 3, the chemical formula of 3-OCA and 5-OCA were further confirmed to be C_16_H_18_O_9_, as indicated by the precursor parent ion at *m*/*z* 353 [M-H]^−^ in negative mode. For 3-OCA, a distinctive peak was identified at *m*/*z* 191, representing the quinic acid fragment ion [M-H-C_9_H_6_O_3_]^−^, resulting from the cleavage of the -COO- ester group and the loss of the caffeoyl fragment. In the case of 5-OCA, the remained peak at *m*/*z* 353 [M-H]^−^ indicated the uncomplete dissociation of the parent ion due to the better chemical stability compared to 3-OCA under similar conditions. In addition to the fragment ion at *m*/*z* 191[M-H-C_9_H_6_O_3_]^−^, a characteristic fragment ion at *m*/*z* 147 [M-H-C_9_H_6_O_3_-CO_2_]^−^ was produced, resulting from further proton rearrangement and cleavage of the deprotonated carboxyl group on the six-membered ring. Furthermore, the fragment ion at *m*/*z* 135 [C_9_H_7_O_3_-CO]^−^ appeared due to the additional cleavage of CO from the caffeoyl fragment. Caffeic acid exhibited a fragment ion at *m*/*z* 163 [M + H-H_2_O]^+^, attributed to the cleavage of H_2_O from the protonated carboxylic group. The fragment ion at *m*/*z* 163 subsequently dissociated to yield the fragment ion at *m*/*z* 145 [M + H-2H_2_O]^+^ through proton transfer and the loss of another H_2_O from the aromatic ring. Additionally, the fragment ion at *m*/*z* 145 produced the fragment ion at *m*/*z* 117 [M + H-2H_2_O-CO]^+^ by the removal of the remaining CO from the original carboxylic group. In addition, the fragment ion at *m*/*z* 163 generated another fragment ion at *m*/*z* 135 [M + H-H_2_O-CO]^+^ by the cleavage of a carbonyl group, as illustrated in Fig. 3.

### 3.5 Anti-glycation assessments of 5-OCA, 3-OCA and caffeic acid

**Fig. 4:**
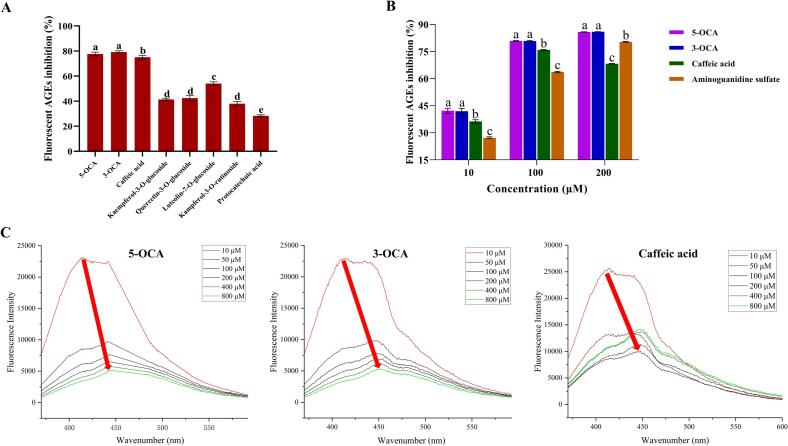
**(A)** The inhibitory effects of eight identified polyphenols (100 μM) on total fluorescent AGEs in BSA-Fru model. **(B)** The inhibitory effects of 5-OCA, 3-OCA and caffeic acid on total fluorescent AGEs in BSA-Fru model. **(C)** The fluorescence spectra of AGEs in the presence of three key polyphenols with different concentrations.

So far, 5-OCA, 3-OCA and caffeic acid have been identified as the key anti-glycation polyphenols in seven different Sakura varieties through multivariate data analysis. The anti-glycation effects of Sakura extracts were confirmed using *in vitro* BSA-Glu/Fru models, with more pronounced effects observed in the BSA-Fru model. Therefore, the inhibitory effects of 5-OCA, 3-OCA, and caffeic acid on AGEs were further validated using the BSA-Fru model, with aminoguanidine sulphate serving as a common positive control (Gao et al., 2024). Fig. 4A demonstrated that 5-OCA, 3-OCA and caffeic acid exhibited the strongest inhibitory effects on the formation of AGEs, consistent with the results from previous multivariant analysis. Fig. 4B illustrated the inhibitive effects of three key polyphenols and positive control on fluorescent AGEs at three concentrations:10 μM, 100 μM and 200 μM. Notably, 5-OCA and 3-OCA demonstrated superior antiglycation effects compared to their metabolite, caffeic acid, and even outperformed the positive control aminoguanidine sulphate across all three tested concentrations. However, no significant difference was observed between 5-OCA and 3-OCA. Caffeic acid exhibited superior anti-glycation activity compared to aminoguanidine sulphate at low and medium concentrations, but unexpectedly demonstrated reduced efficacy at a high concentration of 200 μM. Notably, another study revealed that the AGEs inhibition ratio of caffeic acid at 200 μM was lower than that of caffeic acid at 50 and 100 μM in the BSA-methylglyoxal model, which aligned with our findings in the BSA-Fru model (Gugliucci et al., 2009). The decreased inhibitory effect of caffeic acid on the formation of AGEs can be attributed to its pro-oxidative activity at elevated concentrations (Halliwell, 2007; Kim et al., 2014). Specifically, at a high concentration of 200 μM, the auto-oxidation of the pyrocatechol moiety of caffeic acid generated quinone derivatives, hydrogen peroxide and hydroxyl radicals. These ROS enhanced glycation by accelerating the formation of Amadori products and advanced glycation end-products through non-enzymatic pathways (Wu et al., 2011). Both 5-OCA and 3-OCA exhibited remarkable anti-glycation activities, demonstrating a favourable dose-effect relationship. It further substantiated that 5-OCA, 3-OCA, and caffeic acid were key polyphenols with excellent anti-glycation effects in Sakura. The superior anti-glycation activities of 5-OCA, 3-OCA, and caffeic acid, in contrast to other identified polyphenols in Sakura, were attributed to three synergistic structural characteristics. First, the presence of a catechol moiety with adjacent aromatic hydroxyl groups significantly enhanced the scavenging efficiency of methylglyoxal, a major precursor of AGEs (Han et al., 2024; Shao et al., 2014). Second, the conjugated α, β-unsaturated system facilitated the nucleophilic trapping of reactive carbonyl species (*e.g.*, methylglyoxal and glyoxal) through mono- or di-adduct formation, while simultaneously blocking lysine and arginine residues on proteins, thereby competitively inhibiting the crosslinking of AGEs (Khan et al., 2020; Nooshkam et al., 2020). Third, the compact molecular dimensions of these compound minimized steric hindrance, allowing for efficient interactions with glycation-prone sites on proteins. Collectively, the synergistic interplay of the catechol moiety, α, β-unsaturated conjugation, and optimal molecular dimensions enhanced the anti-glycation efficacies of 5-OCA, 3-OCA, and caffeic acid.

In recent years, fluorescence spectroscopy has become widely used for probing microenvironmental changes in amino acid residues of proteins induced by ligand binding, including interactions with polyphenols and sugars (Cao et al., 2023). Notably, the exceptional sensitivity of tryptophan (Trp) residues to microenvironmental variations makes them ideal probes for monitoring protein conformational changes *via* the intrinsic fluorescence (Qin et al., 2021; Sobhy et al., 2020). In this study, 5-OCA, 3-OCA, and caffeic acid exhibited significant fluorescence quenching effects on glycated BSA, indicating direct interactions with the protein. As illustrated in Fig. 4C, both 5-OCA and 3-OCA demonstrated pronounced concentration-dependent quenching, while caffeic acid exhibited weaker quenching efficacy, suggesting structural specificity in binding affinity. It was noteworthy that the red shifts in spectra for three polyphenols implied potential alterations in the secondary structure of BSA and the exposure of Trp residues due to the alteration of environmental polarity. However, glycation of BSA predominantly targets lysine/arginine residues, as well as the N-terminus (Nisar et al., 2022). Future research should incorporate molecular docking studies to elucidate the binding sites and structural modulation resulting from direct inhibition of AGEs.

### 3.6 Quantitative analysis of 5-OCA, 3-OCA and caffeic acid in seven Sakura varieties

**Fig. 5:**
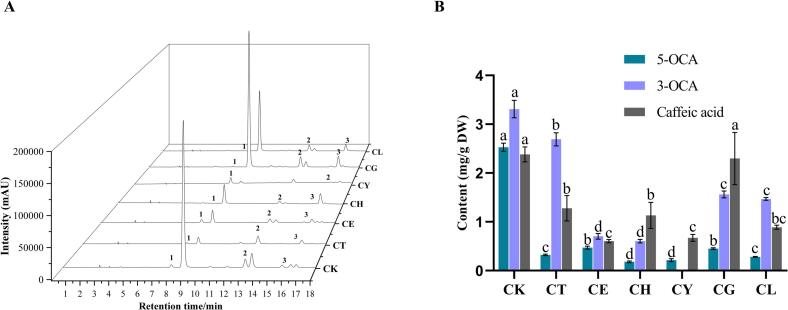
**(A)** HPLC chromatograms of 5-OCA (**1**), 3-OCA (**2**) and caffeic acid (**3**) in seven Sakura extracts. **(B)** Quantitative analysis of three key polyphenols in in seven Sakura varieties.

At present, 5-OCA, 3-OCA, and caffeic acid were identified as the primary anti-glycation polyphenols in Sakura extracts. To corroborate preliminary metabolic profiling data, systematic quantitative analysis was performed across seven Sakura varieties. As illustrated in Fig. 5, HPLC chromatographic profiles and corresponding quantitative results demonstrated significant interspecific variability in these compounds. As expected, the concentrations of 5-OCA, 3-OCA and caffeic acid were the highest in CK, which were 2.52 ± 0.08, 3.31 ± 0.18 and 2.38 ± 0.15 mg/g dry flower (DW), respectively. However, 3-OCA was undetected in CY. Notably, it was found that 3-OCA and 5-OCA were identified as the major phenolic acids with reported concentrations of 1.70 ± 0.25 and 0.47 ± 0.02 mg/g DW in CK in another study, which were lower than our quantitative results (Sola et al., 2022). This discrepancy may be attributed to the methodological differences in preparation of extracts, particularly our implementation of ultrasonic-assisted extraction, which enhanced the extraction efficacy of Sakura polyphenols (Kumar et al., 2021). The content of 5-OCA and 3-OCA in the other six varieties was significantly lower than in CK, while the level of caffeic acid in CK and CG was significantly higher than that in other varieties (*p* < 0.05). It was reported that 3-OCA was identified as the predominant phenolic compound (1.94 ± 0.04 mg/g DW) in *Prunus padus* L. flowers (*Rosaceae* family), which aligned with our observations regarding the predominance of caffeic acid and its derivatives (Olszewska & Kwapisz, 2011). Consequently, these quantitative validations reinforce the reliability of our findings from earlier anti-glycation assessments and multivariate statistical analysis.

### 3.7 Chemical transformation of 5-OCA, 3-OCA, and caffeic acid during simulated *in vitro* digestion

**Fig. 6:**
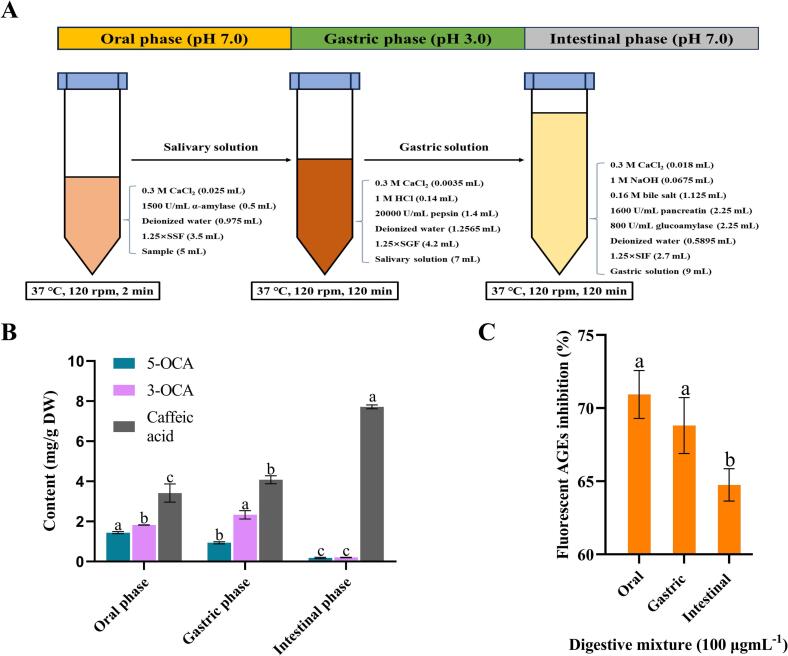
**(A)** Experimental scheme of simulated *in vitro* digestion of CK extract. **(B)** Quantitative analysis of 5-OCA, 3-OCA and caffeic acid at different stages. **(C)** The inhibitory effects of digesta from different stages on total fluorescent AGEs in BSA-Fru model.

**Fig. 7:**
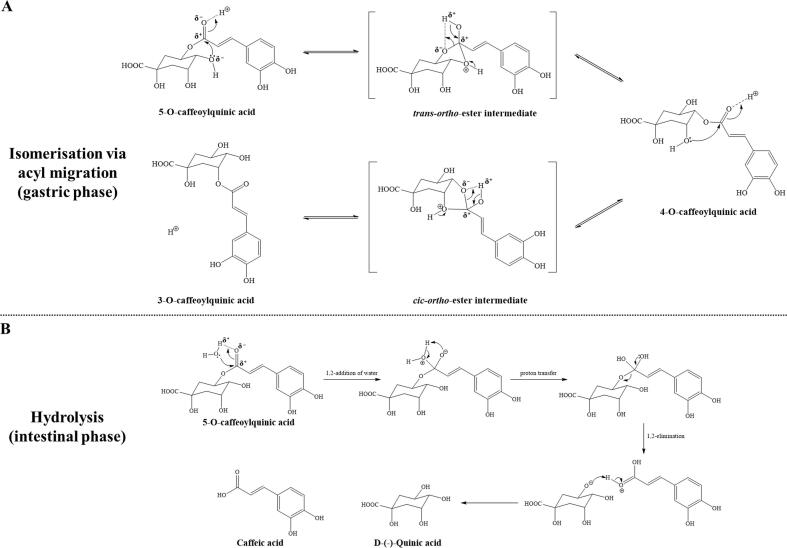
**(A)** The hypothesized mechanisms of isomerization between 5-OCA and 3-OCA in gastric phase. **(B)** The hypothesized mechanisms of 5-OCA metabolized to caffeic acid in intestinal phase.

The increase in research aimed at the study of polyphenolics present in edible resources and their associated health properties marked a significant turning point in the field of food science. To ensure the benefits linked to polyphenolic consumption, it is crucial that bioactive polyphenols found in food matrices, or their metabolites produced during ingestion, are bio-accessible for absorption (Gonçalves Santana et al., 2024). *In vitro* simulated digestion systems, recognized as efficient alternatives to *in vivo* assays, are frequently employed. The standardized *in vitro* digestive model utilized in this study simulated the chemical conditions to which polyphenols were typically exposed throughout the digestive tract by replicating mouth, gastric and intestinal human digestion (Vilas-Boas et al., 2020). Consequently, CK was selected as the most promising variety to investigate how 5-OCA, 3-OCA and caffeic acid dynamically changed during digestion. Fig. 6B presented the quantitative results of the three key polyphenols at each stage of simulated digestion. Interestingly, the concentration of 5-OCA decreased, while the concentration of 3-OCA increased during the gastric phase compared to the undigested sample. Furthermore, the content of caffeic acid did not exhibit significantly changes during the gastric phase. It was hypothesized that 5-OCA underwent isomerization to 3-OCA *via* intramolecular acyl migration under acidic gastric condition, in line with previous findings that 5-CQA was converted to 3- and 4- isomers after 2 h of incubation under alkaline conditions (Matei et al., 2012). Fig. 7A illustrated the potential isomerization of 5-OCA to 3-OCA during simulated gastric digestion. Acyl migration is a chemical process in which an acyl group R-C(=O)- relocated from one position to another intramolecularly. Under acidic conditions, 5-OCA was initially protonated, followed by an electrophilic attack by the lone pair electrons from the ortho hydroxyl group on the electropositive carbonyl carbon. This interaction resulted in the formation of an unstable trans-ortho-ester intermediate. Subsequently, 4-O-caffeoylquinic acid (4-OCA) was formed after proton transfer and acyl group migration. Direct acyl migration from the hydroxyl group on the 5′ carbon to form 3-OCA was hindered by steric constraints. Afterwards, 4-OCA underwent similar electron transfer to produce a cis-ortho-ester intermediate, which was then converted to 3-OCA as the final product through another acyl migration. Notably, the *cis-ortho*-ester intermediate and *trans-ortho*-ester intermediate could not be directly detected by HPLC due to their high instability, which led to the immediate formation of stable products, such as caffeoylquinic acid isomers, during the reversible isomerization process. Furthermore, it has been reported that [^14^C]-quinic acid was used to investigate the intermolecular or intramolecular acyl migration of cinnamoylquinic acid. It was observed that no radioactivity was detected in the ester fraction with the entirety of the recovered radioactivity associated with free quinic acid (Xie et al., 2011). This finding validated that the isomerization *via* migration was an intramolecular process. The proposed isomerization mechanism provided a plausible explanation for the observed decrease in 5-OCA content and the corresponding increase in 3-OCA content during the gastric phase.

As polyphenolics entered the intestinal phase, the digestive environment shifted, accompanied by a gradual increase in pH. Both 5-OCA and 3-OCA nearly disappeared during the intestinal simulation, while the content of caffeic acid increased significantly (*p* < 0.05). This observation was reasonable, as 5-CQA and 3-CQA were prone to hydrolysis under intestinal conditions, resulting in the cleavage of ester group and the subsequent release of caffeic acid and quinic acid, in addition to undergoing isomerization (Farrell et al., 2011). Notably, the increase in caffeic acid exceeded the combined decrease in 5-OCA and 3-OCA, which could be attributed to the metabolism of other polyphenols containing caffeoyl groups present in the CK extract. Fig. 6C indicated that the inhibitory effects of oral and gastric solutions were similar to those of the undigested extracts but were stronger than those of the intestinal solutions, aligning with previous results. The specific mechanism of hydrolysis was illustrated in Fig. 7B. Initially, the electrophilic carbonyl carbon favoured to be attacked by the lone pair of elections on the oxygen atom of the water molecule, followed by the formation of a highly unstable tetrahedral diol intermediate *via* 1,2-addition and proton transfer. The reactive intermediate tended to collapse *via* 1,2-elimination, leading to the release of caffeic acid and quinic acid (Deshpande et al., 2014). Notably, it was reported that quinic acid, classified as a cyclohexane-carboxylic acid, did not exhibit a significant inhibitory effect on protein glycation, attributed to its lack of the conjugated aromatic structure (Bousová et al., 2005). Therefore, although quinic acid was considered a metabolite of 3-OCA and 5-OCA, it was not the focus of this study and was consequently excluded from quantitative analysis.

## 4 Conclusion

This study systematically evaluated the anti-glycation activities of polyphenols across seven Sakura varieties, with a particular emphasis focus on the anti-glycation potential of CK. The metabolic profiles were analyzed using UPLC-MS/MS, revealing that 5-OCA, 3-OCA, and caffeic acid are the primary bioactive components exhibiting anti-glycation effects in Sakura. Further validation through the BSA-Glu/Fru model confirmed the dominant role of these compounds in inhibiting the formation of AGEs, with CK demonstrating particularly strong performance across different glycation models. Additionally, an *in vitro* simulated digestion study indicated that 5-OCA partially isomerized to 3-OCA during gastric digestion, while caffeic acid was identified as the main metabolite in the small intestinal phase.

The innovation of this study lies in its systematic comparison of the anti-glycation effects of different Sakura varieties and a detailed exploration of the transformation and metabolic pathways of key polyphenols during digestion. Given the close association between the formation of AGE and various chronic diseases, the anti-glycation properties of CK and its active components offer new opportunities for the prevention and treatment of these related disorders. However, the anti-glycation efficacies of Sakura extracts and polyphenols were primarily assessed in an *in vitro* BSA-Glu/Fru model, which did not fully replicate the complexities of endogenous glycation and digestion processes in living systems. Therefore, future research should further investigate the bioavailability and efficacy of these compounds *in vivo*, as well as evaluate their safety over long-term consumption. In addition, *in vivo* experiments and clinical trials based on these findings will be crucial for verifying their practical efficacy and application potential. In conclusion, this study provides a theoretical foundation for the application of Sakura in the development of functional foods and pharmaceuticals with promising anti-glycation efficacy.

## CRediT authorship contribution statement

**Yuhang Zhu:** Writing – review & editing, Writing – original draft, Validation, Methodology, Investigation, Formal analysis, Conceptualization. **Zhangtie Wang:** Writing – review & editing, Validation, Investigation. **Xiang Li:** Software, Data curation. **Siyu Chen:** Resources, Investigation. **Daoxin Dai:** Resources, Investigation. **Weihu Li:** Resources, Investigation. **Binhai Shi:** Resources, Investigation. **Baolong Wang:** Resources, Investigation. **Guoliang Jie:** Resources, Investigation. **Baiyi Lu:** Writing – review & editing, Supervision, Resources, Project administration, Methodology, Funding acquisition, Conceptualization.

## Declaration of competing interest

The authors declare that they have no known competing financial interests or personal relationships that could have appeared to influence the work reported in this paper.

## Data Availability

Data will be made available on request.

## Appendix A Supplementary data

Supplementary material: Table S1. The appearance and supplier information of seven Sakura varieties. Image 1
