# Supplementary material for: Identification of key anti-glycation polyphenols in Sakura through metabolic profiling and in vitro assessments
Source: Food Chem X. 2025 Mar 25;27:102416. doi: 10.1016/j.fochx.2025.102416 (PMC11994908; doi:10.1016/j.fochx.2025.102416)
Supplement: Supplementary file 1 — Supplementary material: Table S1. The appearance and supplier information of seven Sakura varieties. [file mmc1.docx]

**Supplementary file**

**Table S1.** The appearance and supplier information of seven Sakura varieties.

| Names | Appearance | Supplier and producing area |
| --- | --- | --- |
| *Cerasus serrulata ‘Kanzan’*  (CK) | 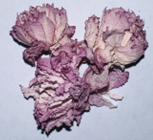 | Supplier: Yangzhou China and Yeal Food Co., Ltd.  Producing area: Ganquan Town, Yangzhou, Jiangsu Province, China |
| *Cerasus yedoensis (Matsum.) T.T. Yu et Li*  (CT) | 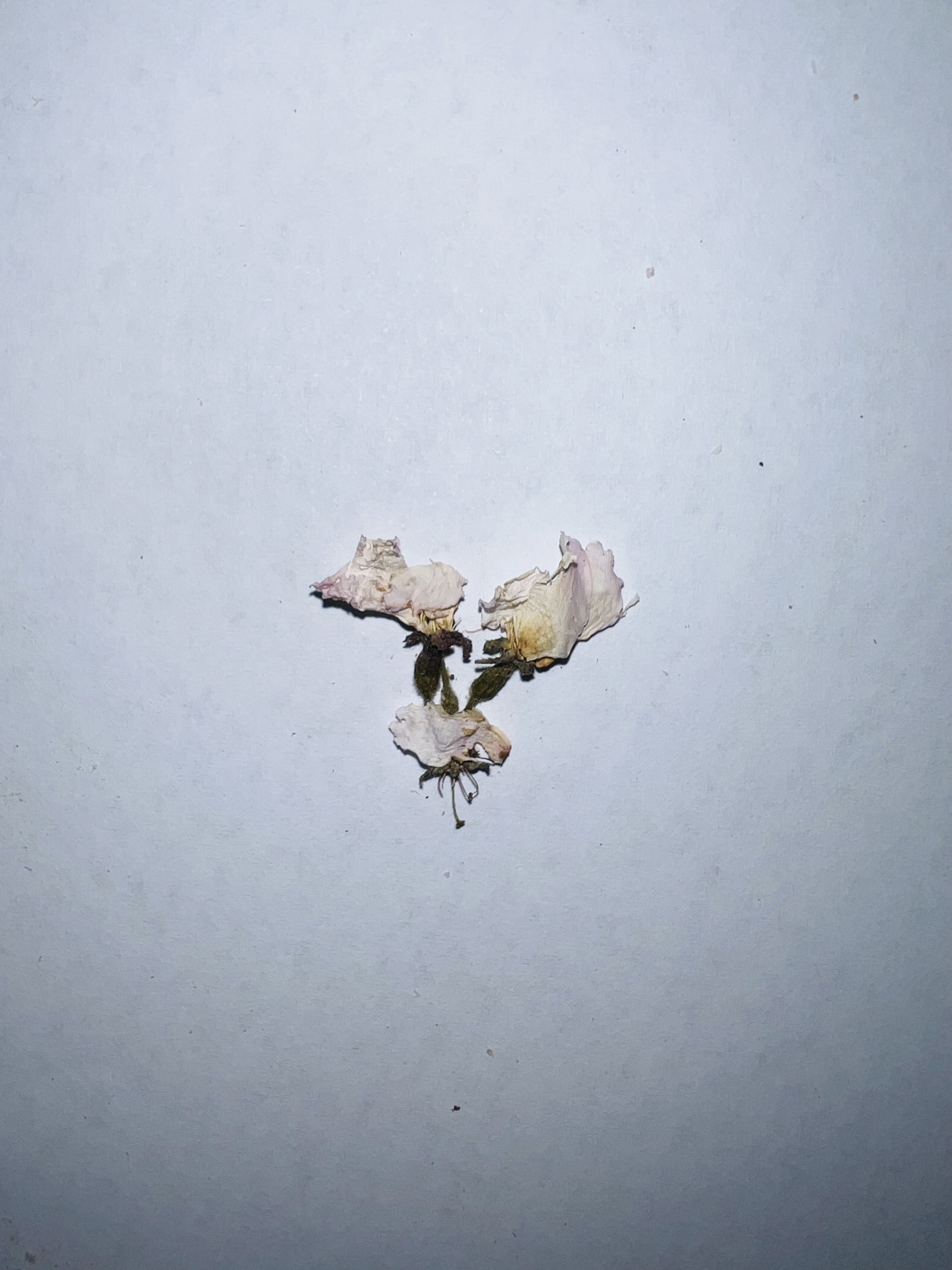 |  |
| *Cerasus sargentii (Rehder) Eremin, Yushev & L. N. Novikova*  (CE) | 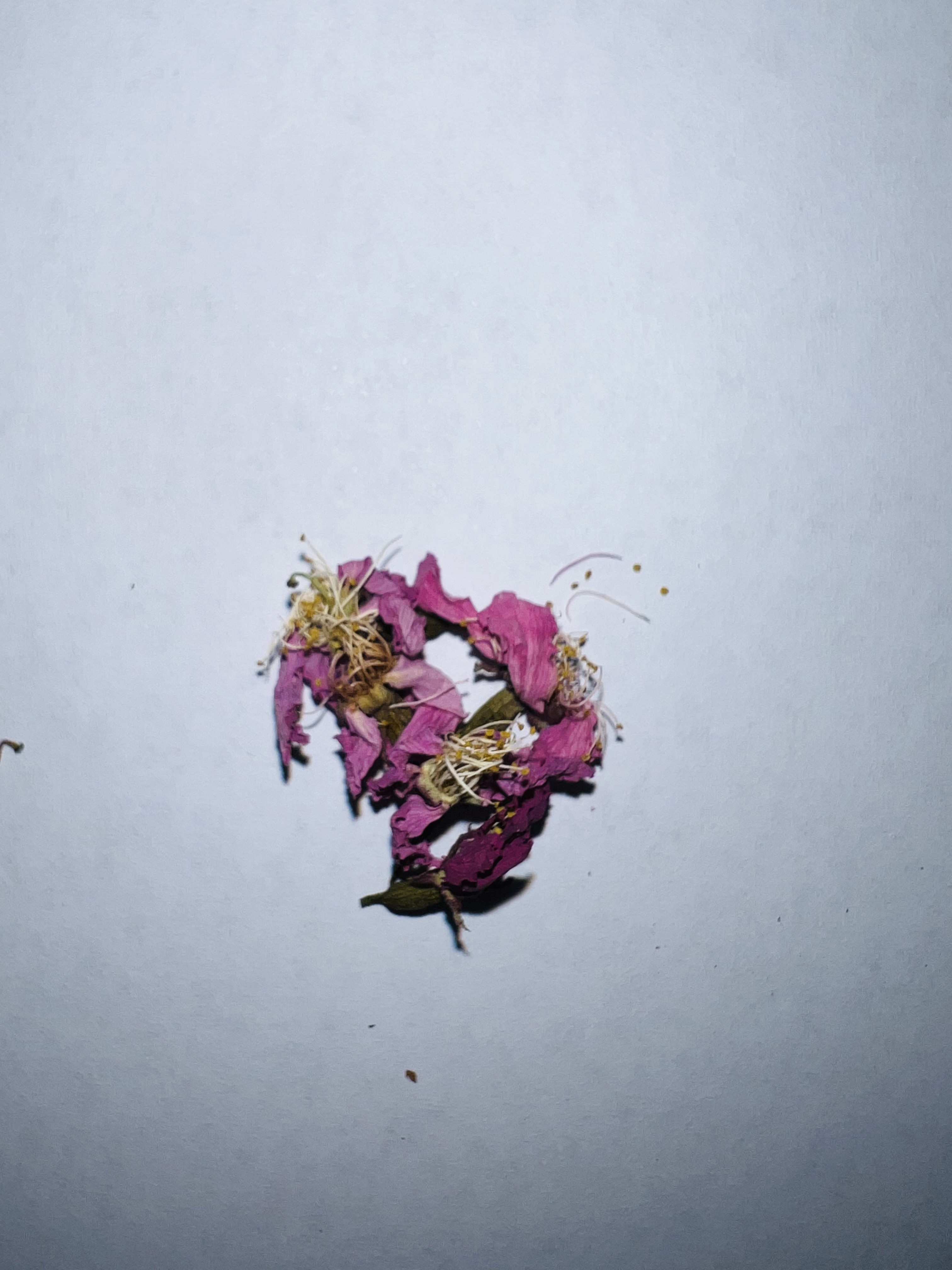 |  |
| *Cerasus speciosa (Koidz.) H. Ohba* (CH) | 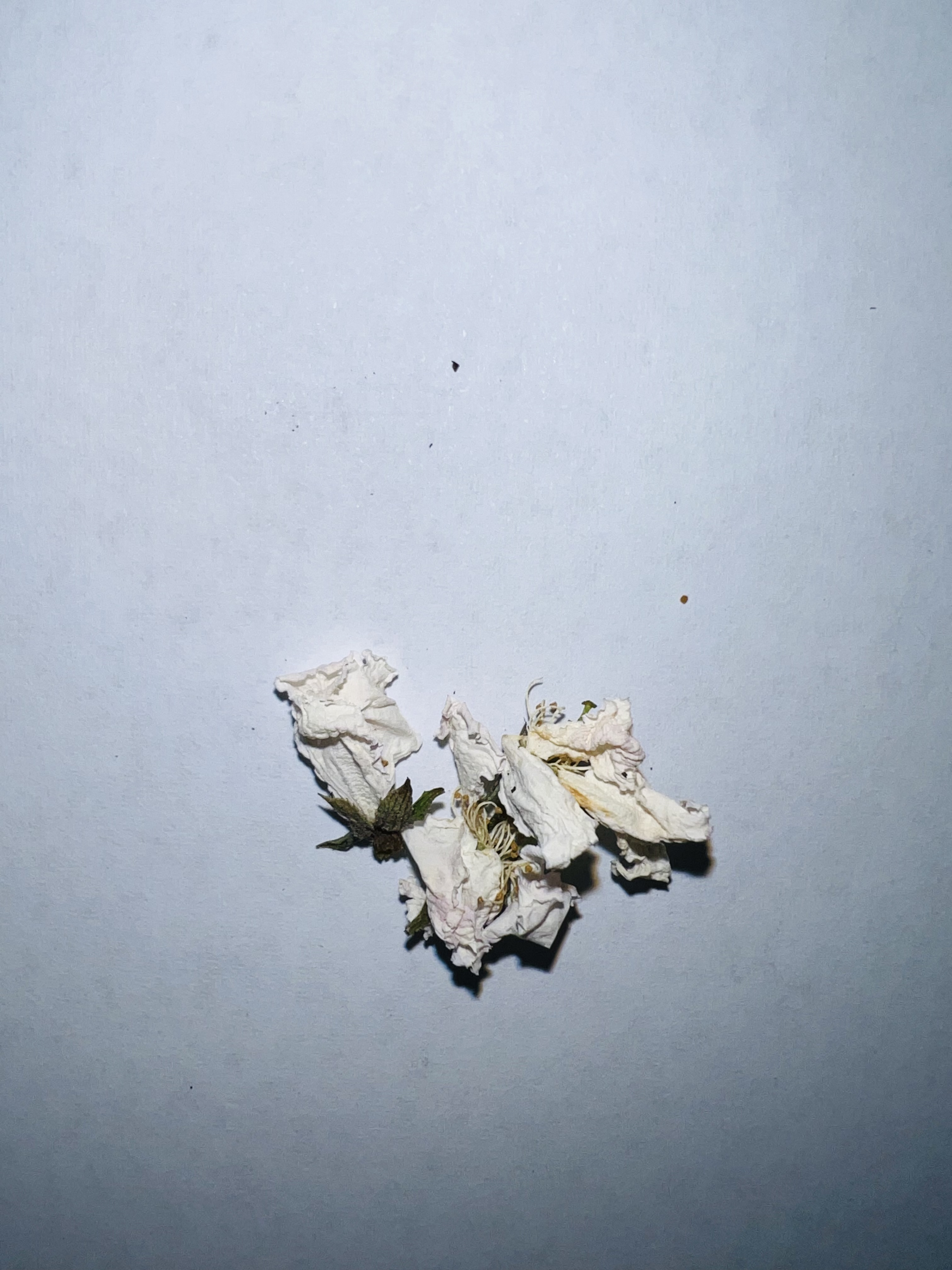 |  |
| *Cerasus campanulata (Maxim.) Yü et Li* (CY) | 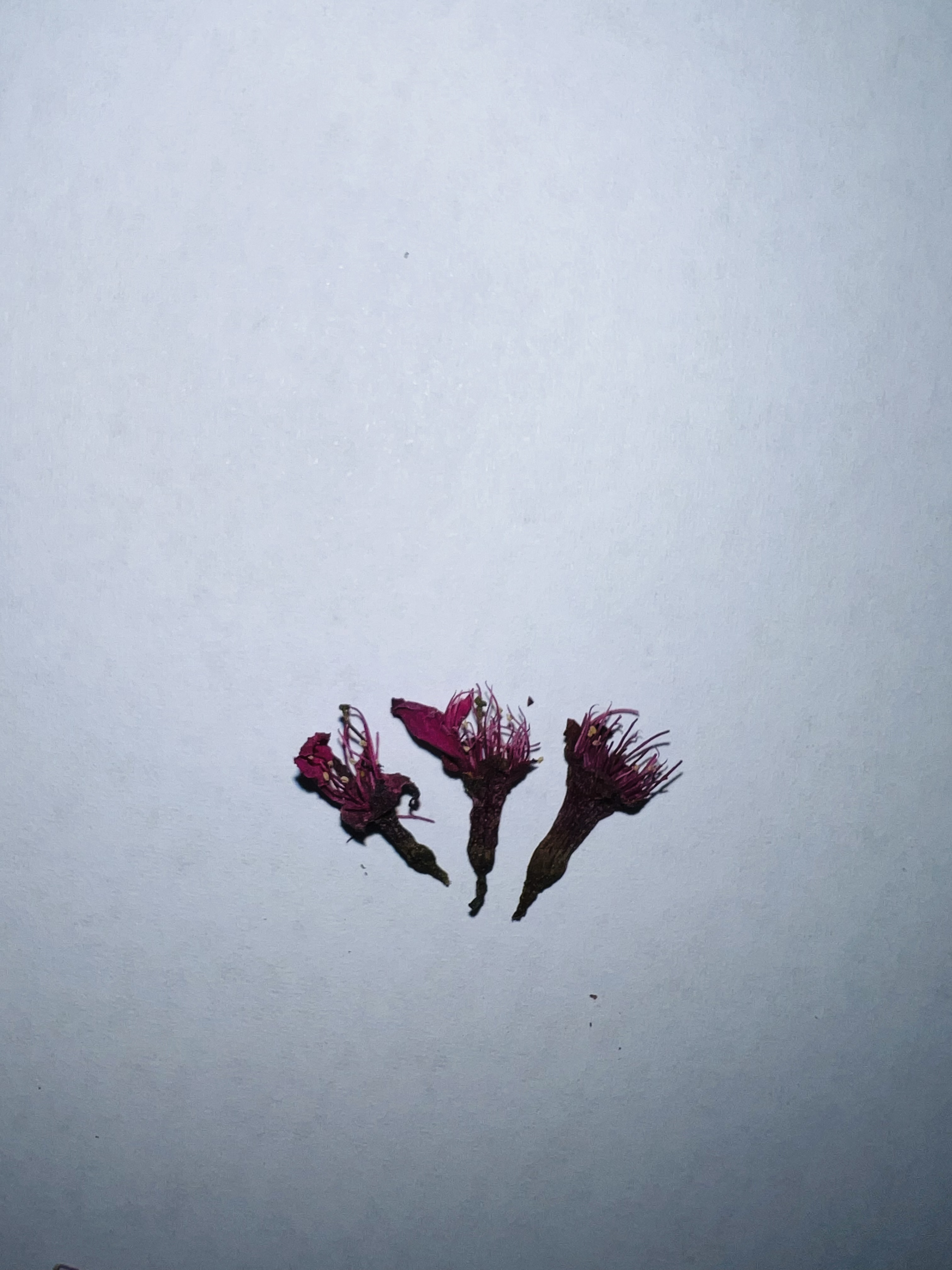 |  |
| *Cerasus serrulata (Lindl.) G. Don*  (CG) | 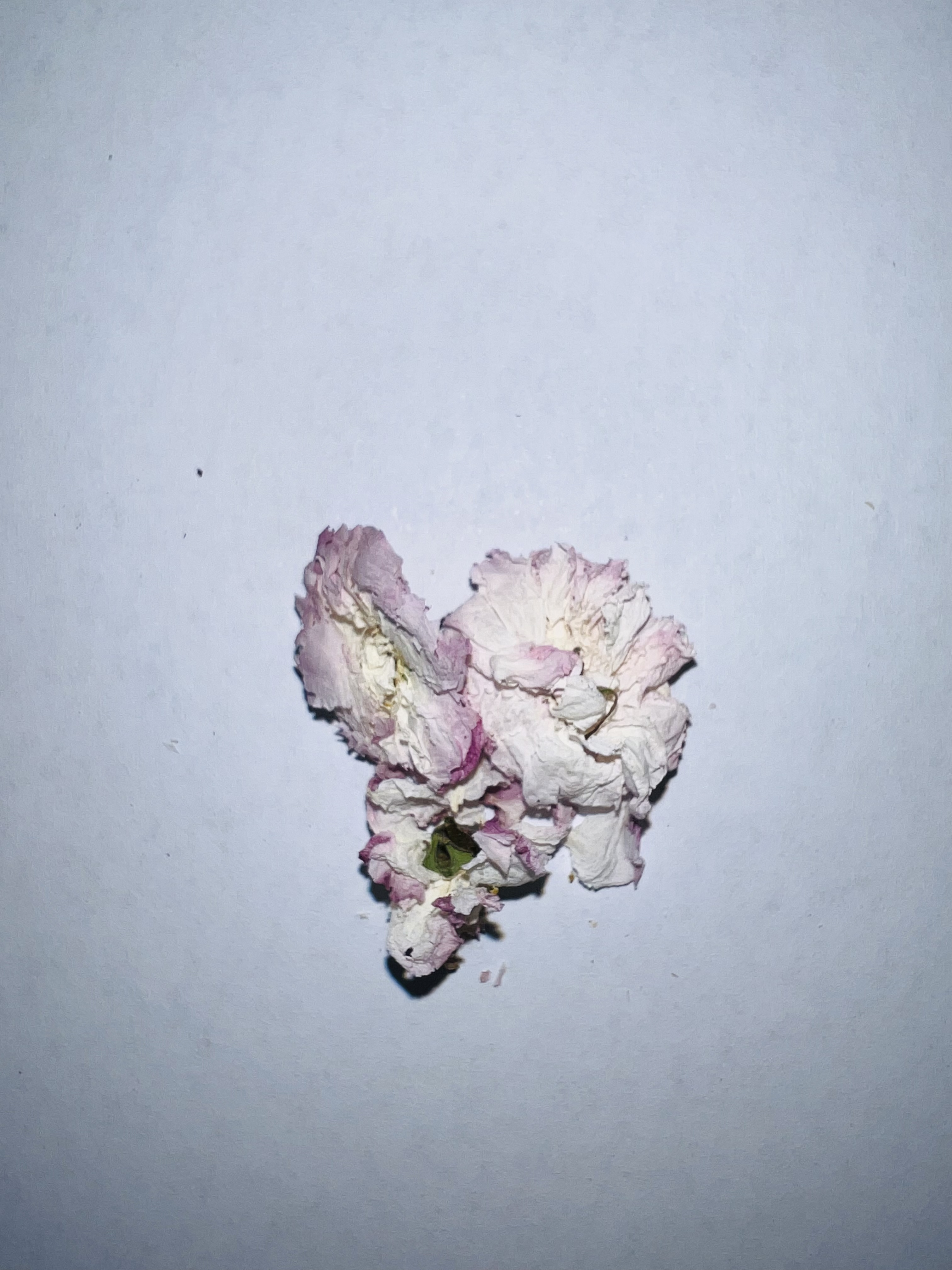 |  |
| *Cerasus serrula (Franch.) Yü et Li* (CL) | 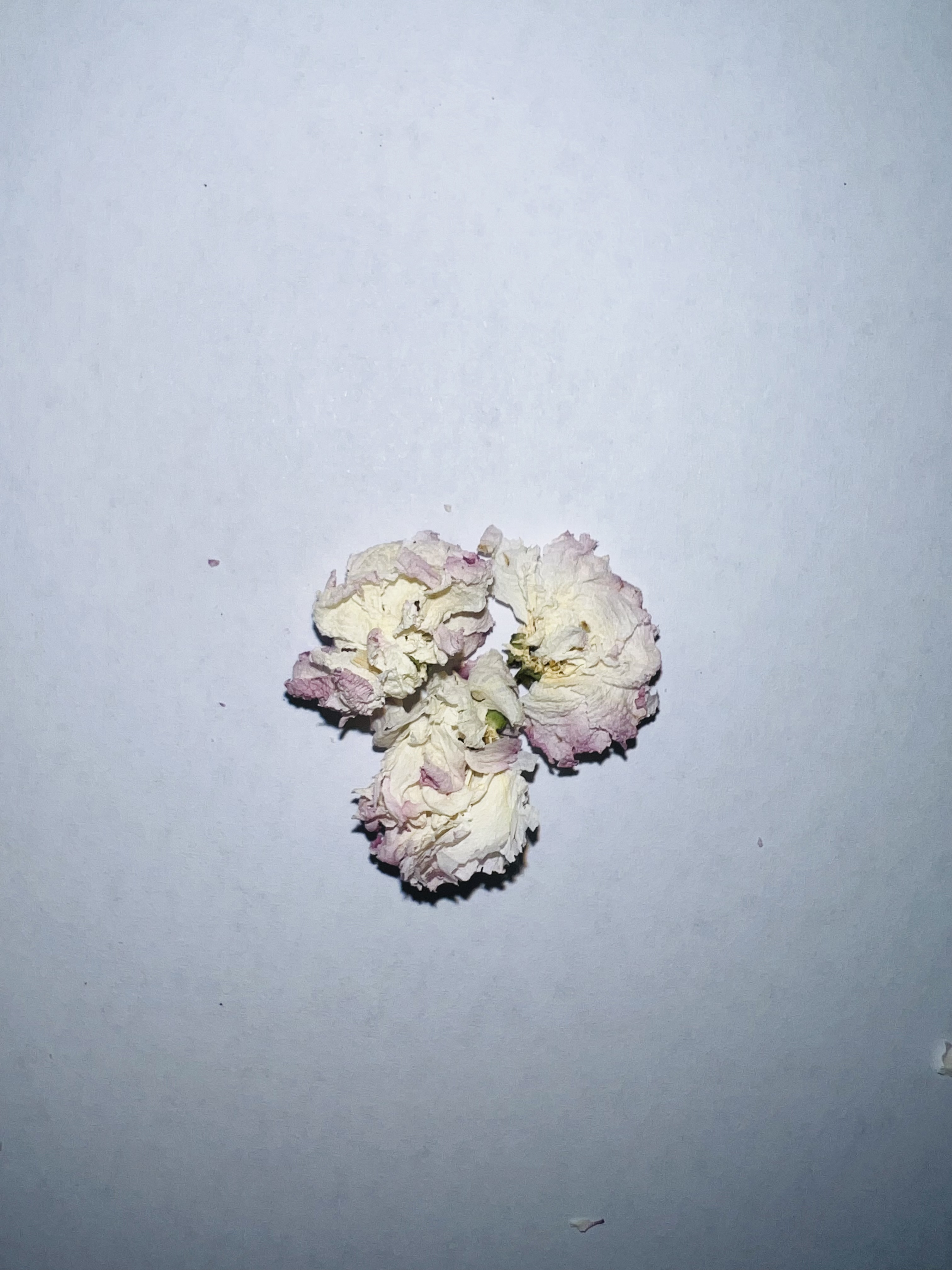 |  |
